# Genetic structuring and fixed polymorphisms in the gene *period* among natural populations of *Lutzomyia longipalpis* in Brazil

**DOI:** 10.1186/s13071-015-0785-6

**Published:** 2015-04-01

**Authors:** César Raimundo Lima Costa, Moises Thiago de Souza Freitas, Carlos Alberto Santiago Figueirêdo, Nádia Consuelo Aragão, Lidiane Gomes da Silva, Carlos Brisola Marcondes, Raimundo Vieira Dias, Tereza Cristina Leal-Balbino, Manuela Barbosa Rodrigues Souza, Marcelo Ramalho-Ortigão, Valdir de Queiroz Balbino

**Affiliations:** Departamento de Genética, Universidade Federal de Pernambuco, Avenida da Engenharia S/N, Cidade Universitária, 50.740-600 Recife, Pernambuco Brazil; Departamento de Microbiologia, Imunologia e Parasitologia, Universidade Federal de Santa Catarina, Campus Reitor João David Ferreira Lima, 88040-900 Florianópolis, Santa Catarina Brazil; Centro de Controle de Zoonoses, Rua Finlândia S/N, Parque Silvana II, 62010-970 Sobral, Ceara Brazil; Departamento de Microbiologia, Centro de Pesquisas Aggeu Magalhaes, Avenida Professor Moraes Rego S/N, Cidade Universitária, 50740-465 Recife, Pernambuco Brasil; Department of Entomology, Kansas State University, W. Waters Hall 123, 66506-400 Manhattan, KS USA

**Keywords:** *Lutzomyia longipalpis* complex, *Period* gene, Fixed polymorphism

## Abstract

**Background:**

Even one hundred years after being originally identified, aspects of the taxonomy of the sand fly *Lutzomyia longipalpis*, the principal vector of *Leishmania infantum* in the Americas, remain unresolved for Brazilian populations of this vector. The diversity of morphological, behavioral, biochemical, and ethological characters, as well as the genetic variability detected by molecular markers are indicative of the presence of a complex of species.

**Methods:**

In this study, a 525 bp fragment of the *period* gene was used to evaluate sympatric populations of *L. longipalpis*. A combination of probabilistic methods such as maximum likelihood and genetic assignment approach to investigate sympatric species of *L. longipalpis* were applied in three populations of Northeast Brazil.

**Results:**

Fixed polymorphisms in geographically isolated populations of *L. longipalpis* from two localities in the state of Ceará and one in the state of Pernambuco, Brazil, was identified in a 525 bp fragment of the gene *period* (*per*). Our results suggest a direct relationship between the number of spots found in males’ tergites and the genetic variation in cryptic species of *L. longipalpis*. The fragment used in this study revealed the nature of the ancestral morphotype 1S.

**Conclusion:**

New polymorphisms were identified in the gene *per* which can be used as a genetic barcode to sympatric taxonomy of *L. longipalpis*. The *per* gene fragment confirmed the presence of two siblings species of *L. longipalpis* in Sobral and showed that these same species are present in two other localities, representing an expansion within the *L. longipalpis* species complex with regards to the states of Ceará and Pernambuco.

**Electronic supplementary material:**

The online version of this article (doi:10.1186/s13071-015-0785-6) contains supplementary material, which is available to authorized users.

## Background

A species complex is generally defined as a group of morphologically similar species that differ with regards to genetic and ethological aspects [1]. Low flight range and geographic isolation between populations are major drivers of allopatric speciation, contributing to constitution of cryptic species in Phlebotomine sand flies [2-4]. The occurrence of cryptic species is a fairly common event in other disease vectors and have been described in *Anopheles gambiae, Culex pipiens*, *Triatoma dimidiata* and phlebotomines [5-8].

*Lutzomyia longipalpis*, the principal vector of *Leishmania infantum*, the etiologic agent of American visceral leishmaniasis*,* has a discontinued distribution throughout the Neotropical region with different populations displaying aspects compatible with those of a species complex [9,10]. The existence of cryptic species in the *Lutzomyia longipalpis* complex was supported by studies using morphological and molecular markers in populations from Central and South America leading to the identification of *L. pseudolongipalpis* [11], formally recognized as the first taxon of the *L. longipalpis* complex (reviewed by [12]). Later, it was also suggested that *L. cruzi* should be regarded as a cryptic species within the *L. longipalpis* complex [13].

In Brazil, the presence of a *Lutzomyia longipalpis* complex was first proposed by Mangabeira [14] and was based on the number of abdominal pale spots, one (1S) or two (2S), visible on abdominal tergites. Analyses of male sexual pheromones and courtship (mating) sounds, as well as microsatellite markers and speciation genes, have provided further evidence of a *L. longipalpis* species complex [10].

The gene *period* (*per*) controls biological rhythms and plays a central role in eclosion and insect locomotion activity [15,16]. *Per* has been used in studies of fruit flies (Drosophilidae) population genetics and has been shown to be a useful marker, especially when it comes to identifying cryptic species [17,18]. In sand flies, *per* has also been utilized in studies of population genetics to identify possible members of the *L. longipalpis* complex in Brazil. Combined with certain behavioral markers (e.g. male mating songs and the types of sexual pheromones produced), analysis of the variability of *per* suggested the existence of two major population groups of *L. longipalpis* in Brazil. One group, with at least five species, is found in most localities where *L. longipalpis* has been reported. This group displays a single spot (1S) on the abdominal tergites, but different pheromones and patterns of pulsating songs. The second group, with two spots (2S), is represented by a single species present in the North, Northeast and Southeast regions of Brazil. Males in this group produce Burst-type songs and the pheromone cembrene-1 [19].

The NE region in Brazil is the region with the highest index of visceral leishmaniasis [20], and studies demonstrated the presence of at least two sister species within *L. longipalpis* complex [19,21,22]. In Sobral, State of Ceará, these species occur in sympatry and can be separated based either on their abdominal spots 1S or 2S, the different mating songs, the types of pheromones, or the genetic composition of males [23]. Hence, genetic markers related to the abdominal spots may indeed be an important tool for molecular taxonomy of the *L. longipalpis* complex, especially concerning eco-epidemiological studies to assess potential vectorial capacity between distinct populations.

Here, we investigated the presence of polymorphisms in the gene *per* and their relatedness to the number of abdominal spots in *L. longipalpis* males. Sand flies from three localities in the NE of Brazil, separated by distances ranging from 108 km to as much as 457 km from each other, were used for our analyses. Four novel fixed single nucleotide polymorphisms (SNP) were identified, some able to separate between the 1S and 2S flies. In addition, our results point to an ancestral origin of the morphotype 1S. This study contributes to the understanding of the natural history of *L. longipalpis* populations and provides new insights about the biogeography of this sand fly.

## Methods

### Field collection and identification of phebotomine sand flies

Sand fly trappings were carried out in Sobral (3°41′15″S; 40°21′5″W) and Caririaçu (07°02′31″S; 39°17′02″W) in the State of Ceará, and in Bodocó (07°46′42″S, 39°56′28″W) in the State of Pernambuco, Brazil. All locations included in this study have a BSw‘h’ climate in accordance to the Köppen climate classification [24], with temperatures ranging from 23°C to 36°C and low annual rainfall (936 mm to 1100 mm). The localities included in this study are fully inserted in Caatinga biome, with vegetation composed mainly of ligneous and herbaceous species with high degree of xerophily [25].

Sand flies were trapped in the surrounding houses and domestic animal shelters using five CDC-type miniature light traps positioned at approximately 0.6 m from the ground. Sand flies were identified according to Young and Duncan [26], and *L. longipalpis* males were separated based on the number of abdominal spots into 1S and 2S.

### DNA extraction, PCR and sequencing

Genomic DNA was extracted from each specimen using 100 μl of Chelex® resin (Bio-Rad, Hercules, CA) based on the protocol described by Solano et al. [27] with modifications. Briefly, each sand fly was homogenized using a hand held homogenizer and pestle in 10% Chelex solution followed by incubation at 56°C for an hour. Sample lysates were incubated at 95°C for 30 min, centrifuged for 6 min at 13,000 × g, and each supernatant was removed and stored at −4°C.

For each DNA sample isolated, a segment of 525 bp of *per* [28] was amplified by PCR using the Master Mix kit (Promega, Fitchburg, WI). PCRs were carried out using a PTC-200 thermocycler (MJ Research, Ramsey, MN) as follows: 5 min at 95°C, and 30 cycles of 95°C for 30 seconds, 50°C for 30 seconds, and 72°C for 1 min, with a final extension at 72°C for 10 min. A 5 μl aliquot from each PCR product was separated on 0.5% agarose and the remainder was purified using the Genomic DNA Purification Wizard kit (Promega, Fitchburg).

Bi-directional sequencing reactions were performed on each purified PCR product using the BygDye Terminator v3.1 Matrix Standard (Applied Biosystems, Foster City, CA) and analyzed using an ABI3100 Sequence Analyzer (Applied Biosystems, Foster City). Each sample was sequenced in duplicate and the sequences obtained were assembled and analyzed with the Staden package [29] based on the values of *Phred* 40 [30]. The high-quality sequences obtained were deposited in GenBank (accession numbers KF479047-KF479163 and KP013849-KP013902).

### Population genetics

Sequence alignment was performed using the program ClustalW (MEGA v.5.1), and the conserved and variables sites (parsimony informative and singletons sites) also were verified using MEGA v.5.1 [31]. The analyses of polymorphisms and the neutrality tests were performed using DnaSP 5.10.1 [32]. For optimal viewing of polymorphisms, parsimony-informative sites were exported to compose sequence logos obtained by Weblogo v. 3.2 [33].

The genetic structuring of the *L. longipalpis* populations was verified using sequences alignment throughout STRUCTURE v.2.3 [34], with model-based approach. This algorithm clusters individuals into populations. The assigned proportion of each individual belonging to each population (membership coefficient Q) was estimated using Bayesian statistics and Markov Chain Monte Carlo simulations (MCMC). MCMC simulations were performed with 100,000 interactions of burn-in period and followed by one million steps. For each value of K (1 to 10), 10 interactions were performed in order to estimate the K values, and the most likely population (or cluster) number was determined by the ΔK analysis [35]. Matched Fst and MN values [36] were obtained through the Arlequin v.3.1 software [37], using 1000 random permutations. Population structure was verified by the utilization of AMOVA [37], based on the evaluation of different hierarchical groups.

### Phylogenetic analysis

For the UPGMA tree, the Fst values were imported into MEGA V. 5.1 software. The jModeltest v.0.1.1 software was used to select the best-fit DNA substitution model for ML analysis based on the Akaike information criterion (AIC) algorithm [38]. The best-fit model for the *per* sequence analyzed was the *TVM + I + G*. The maximum likelyhood (ML) tree for *per* sequences was calculated using PhyML v. 3.0 [39].

## Results

**Figure 1:**
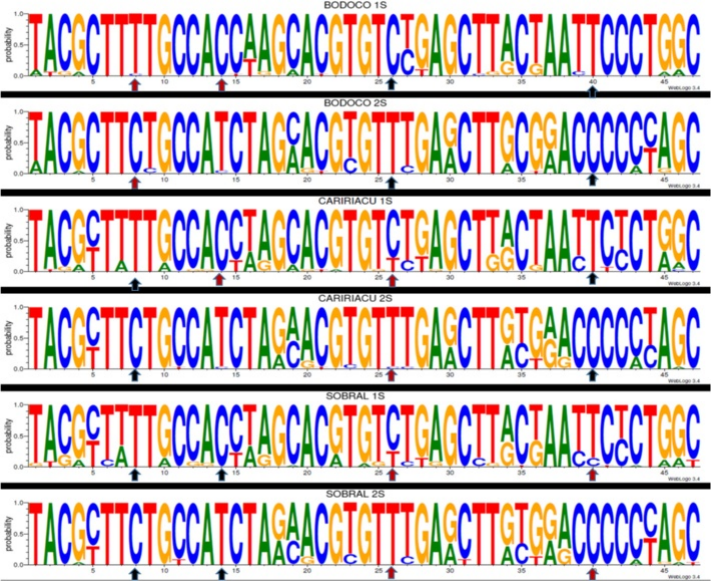
**Schematic representation of polymorphisms of a fragment of 525 bp of the gene** ***period*** **using Weblogo [** 33 **].** Shown are the sequences obtained from *L. longipalpis* collected in Bodocó, State of Pernambuco (Bodocó 1S and Bodocó 2S) and Caririaçu and Sobral, State of Ceará, Brazil. Font size is indicative of the frequency of a nucleotide at any given site. Fixed (black arrows) and partially fixed (red arrows) SNPs are indicated.

Following identification and sequencing of the sand flies collected, we obtained a total of 171 sequences of the gene *per* from *L. longipalpis* males, being 58 (35 1S and 23 2S) from Sobral, 59 (28 1S and 31 2S) from Caririaçu, and 54 (30 1S and 24 2S) from Bodocó. Tests such as Tajima’s neutrality, haplotype diversity, nucleotide diversity, and average number of nucleotide differences for each population studied are summarized in Table 1. Analysis of the parsimony informational sites identified four fixed single nucleotide polymorphisms (SNP) within the 525 bp fragment of *per* used in our analyses. All SNPs can be utilized to separate phenotypes. The SNP identified on nucleotide position T124C (the first and second nucleotides occur in 1S and 2S phenotypes, respectively) located within exon 1, is fixed in *L. longipalpis* from both localities in Ceará. The SNP identified in position C424T (within exon 2) is fixed in the *L. longipalpis* from Bodocó and Caririaçu. The SNP at position C171T also separates the two phenotypic forms (1S and 2S) and is fixed in *L. longipalpis* from Sobral only. In contrast, the SNP at position T256C, which also separates 1S from 2S, is present in the *L. longipalpis* from Bodocó only (Figure 1).

**Table 1:** **Neutrality tests and intra-population genetic diversity measures for each sample**

**Samples**	**Tajima’s** ***D***	**N**	**Hd**	***π*** **± SE**	**NS**	**h**	**k**
Bodocó 1S	−1.00443	30	0.959	0.00684 ± 0.00094	20	20	3.593
Bodocó 2S	−0.48148	24	0.986	0.00885 ± 0.00089	20	21	4.649
Caririaçu 1S	−0.30235	28	0.997	0.01392 ± 0.00108	31	27	7.310
Caririaçu 2S	0.37792	31	0.994	0.01058 ± 0.00056	20	28	5.553
Sobral 1S	0.17037	35	1.000	0.01648 ± 0.00108	34	35	8.652
Sobral 2S	0.46447	23	0.996	0.01162 ± 0.00089	20	22	6.103
Total	−0.05022	171	0.997	0.01901 ± 0.01901	55	149	9.980

Tajima's D; Tajima test of neutrality; N: sample size; HD: Haplotypic diversity*; π* ± SE: nucleotide diversity and standard errors (SE); NS, Number of polymorphic sites; h, Haplotypes; K, average number of nucleotide differences. *p,0.05.

**Figure 2:**
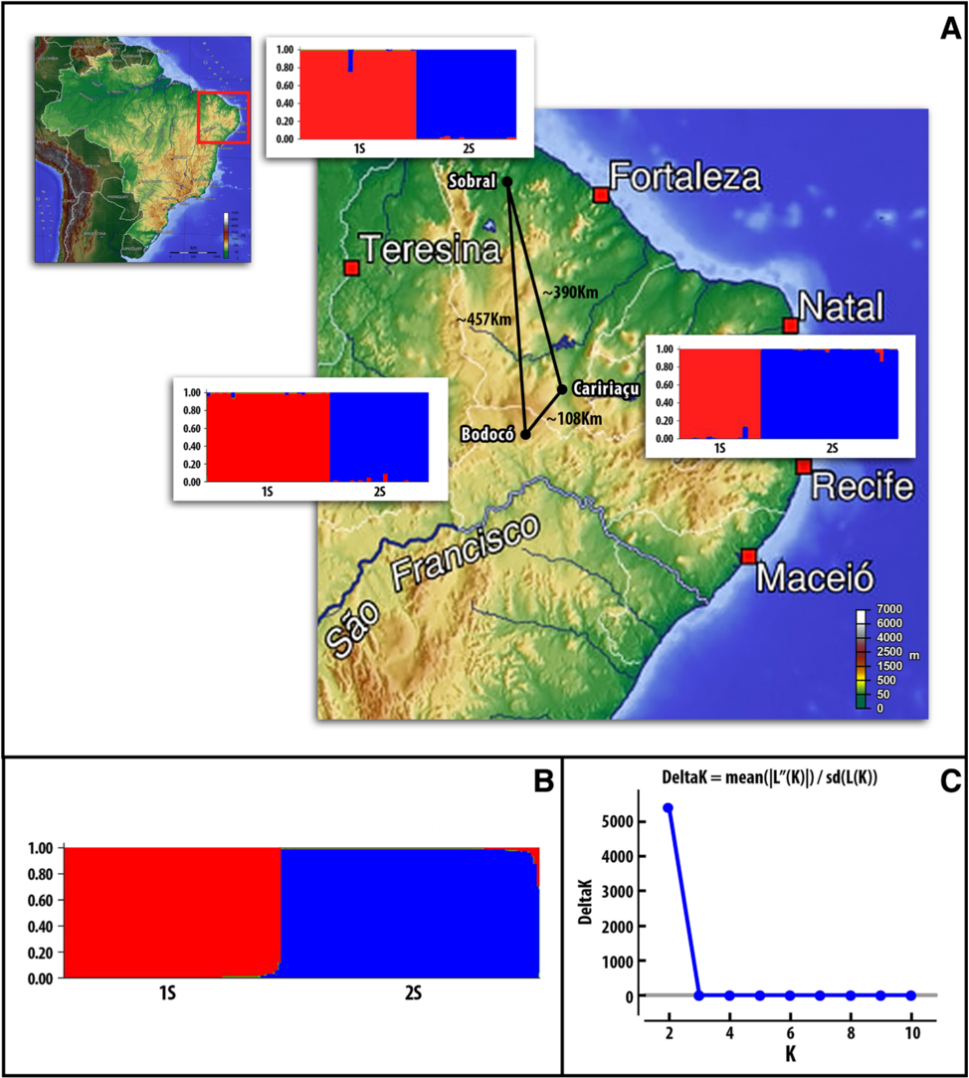
**Elevation map and genetic assignment analysis. A**: Relief map of Brazil (inset) and the distance between localities of Bodocó, Caririaçu and Sobral, which exhibits a very rugged topography ranging from 63 m to 900 m of altitude. The bar plots generated by the software STRUCTURE inferred the genetic structure of populations of *L. longipalpis* from each places studied. **B**: Genetic assignment from all localities, specimens 1S phenotype were assigned to the red group, and 2S specimens were assigned to the blue group. **C**: Delta K method [35] indicating the presence of two genetic populations.

In the genetic assignment analyses of *L. longipalpis*, with each population assessed separately, two distinct genetic groups associated with abdominal spots were observed and each sequence possessing a probability (Q) greater than 80% to belong to each genetic group (Figure 2A). When sequences from all three places were combined, the genetic assignment test indicated the presence of two genetic groups associated with 1S and 2S morphotypes, as suggested by the peak of Δk indicating the presence of two genetically distinct populations (Figure 2B and 2C, respectively).

**Figure 3:**
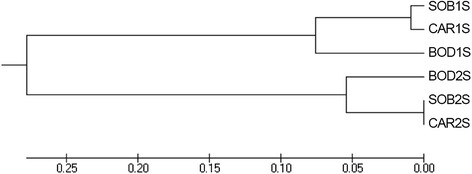
**AMOVA UPGMA tree for populations of** ***L. longipalpis*** **.** UPGMA tree constructed from the Fst values for each phenotype and their respective localities. Populations of Sobral and Caririaçu 1S and 2S (SOB1S, SOB2S, CAR1S and CAR2S) are separate populations of Bodocó (BOD1S and BOD2S).

Similarly, when Fst was used to verify the genetic structuring in the three localities (Bodocó, Caririaçu and Sobral), the presence of two morphotypes related to 1S and 2S were again observed. The *L. longipalpis* 1S populations of Bodocó, Caririaçu and Sobral display low Fst values when compared to each other, forming a genetically similar group (Group 1S). A similar pattern was observed when comparing the 2S populations amongst themselves. However, the Fst value were the highest when comparing different phenotypes, even from the same location (Table 2). The tree of distances obtained from Fst values exhibited a phylogeographic profile with Bodocó population more distant from Ceará populations, when comparing pairs of phenotypes (Figure 3). Utilizing these groups, AMOVA showed significantly the highest percentage of variation between 1S and 2S groups (Table 3).

**Table 2:** **Genetic differentiation among samples**

**Populations**		***F***_**st**_	**Nm**	**Dxy**	**Da**	**Ss**	**Sf**
Sobral 1S	Caririaçu 1S	0.01892	2.86330	0.01549	0.00029	26	0
Sobral 1S	Sobral 2S	0.50016	2.73255	0.02787	0.01382	11	2
Sobral 1S	Caririaçu 2S	0.48584	0.25225	0.02611	0.01258	9	1
Caririaçu 1S	Sobral 2S	0.53515	0.27071	0.02723	0.01445	9	3
Caririaçu 1S	Caririaçu 2S	0.52220	0.28603	0.02542	0.01317	9	2
Sobral 2S	Caririaçu 2S	0.00000	27.22473	0.01097	0.00000	16	0
Bodocó 1S	Sobral 1S	0.15423	0.51128	0.01379	0.00213	16	0
Bodocó 1S	Caririaçu 1S	0.15249	0.52877	0.01225	0.00187	16	0
Bodocó 1S	Bodocó 2S	0.66122	0.54558	0.02317	0.01532	8	2
Bodocó 1S	Sobral 2S	0.64012	0.41809	0.02566	0.01642	7	3
Bodocó 1S	Caririaçu 2S	0.63698	0.44627	0.02399	0.01528	6	1
Bodocó 2S	Sobral 1S	0.50950	0.46142	0.02583	0.01316	7	1
Bodocó 2S	Caririaçu 1S	0.54926	4.08197	0.02527	0.01388	8	2
Bodocó 2S	Sobral 2S	0.10863	4.21053	0.01149	0.00125	12	0
Bodocó 2S	Caririaçu 2S	0.10793	inf	0.01089	0.00118	12	0

*F*_st_: pair-wise genetic differentiation; Nm: number of migrants per generation; Dxy: average number of nucleotide substitutions per site between populations; Da: number of net nucleotide substitutions per site between populations; Ss: number of shared polymorphisms between pairs of population; Sf: number of fixed differences between pairs of populations.

**Table 3:** **AMOVA results for** ***L. longipalpis*** **populations**

**Source of variation**	**d.f.**	**Percentage of variation**
Among groups	1	50.69
Among populations within groups	4	4.28
Within populations	165	45.04
Total	170	
F_SC_ (haplotypes/populations within groups)		0.08672
F_ST_ (haplotypes/populations/groups)		0.54963
F_CT_ (populations/groups)		0.50687

Phenotype Groups: 1S (Bodocó 1S, Caririaçu 1S and Sobral 1S), and 2S (Bodoco 2S, Caririaçu 2S and Sobral 2S).

**Figure 4:**
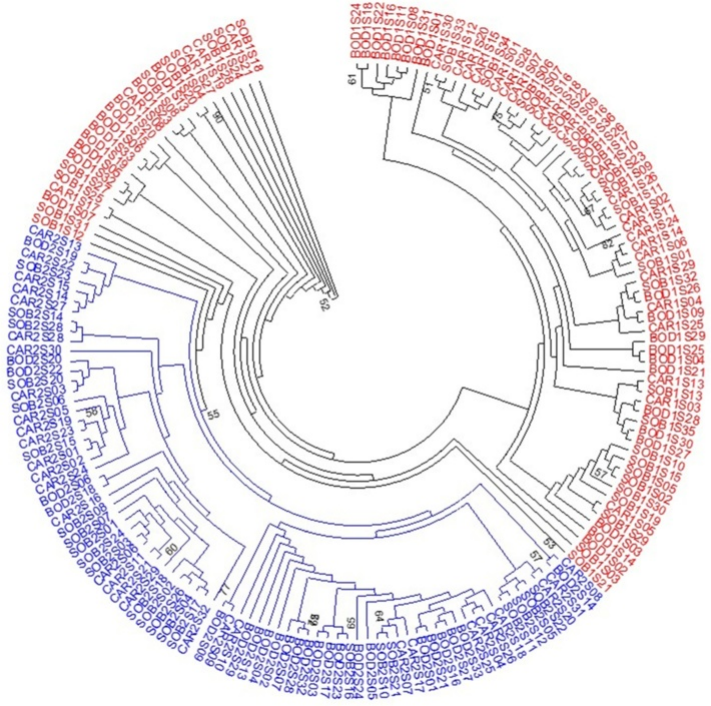
**Maximum likelihood tree obtained of the** ***TVM + I + G*** **model has shown the results of using 525 bp from** ***Lutzomyia longipalpis*** **period marker.** The localities of Bodocó (BOD), State of Pernambuco and Caririaçu (CAR) and Sobral (SOB), State of Ceará, Brazil. The topology shown (55% bootstrap support) consistently separates the two morphological variants known for *L. longipalpis*, the morphotypes 1S and 2S, as well as puts in evidence the derived monophyletic position of the 2S group.

The maximum likelihood tree showed a geographical separation between taxons. Although, the phenotypic groups were separated, indicating an ancestral nature of the 1S clade, supported by the monophylic nature of the 2S group with 55% bootstrap value (Figure 4).

## Discussion

The composition of the *L. longipalpis* complex is still a rather controversial topic even though the distribution of this sand fly in Brazil strongly suggests the presence of at least five species as part of this complex [10,19].

Our assessment of polymorphism frequency in the *per* gene in different populations of *L. longipalpis* revealed for the first time the presence of two fixed polymorphisms (124 and 424) that can reliably be used to separate 1S and 2S phenotypes. The SNP at position T124C was previously reported in *L. longipalpis* populations collected in the town of Jaíba, Minas Gerais, located 1,347 km from Sobral and 1,040 km from Caririaçu [19]. The SNP at position C424T was identified in *L. longipalpis* populations from Bodocó and Caririaçu. Two additional SNPs, at positions C171T and T256C can be used as coadjuvants to separate between the 1S and 2S *L. longipalpis* morphotypes from Sobral and Bodocó, respectively. Although all polymorphisms are synonymous, the combined SNPs can be used as markers for cryptic species in *L. longipalpis*.

The importance of the ML method for the detection of recent speciation events also was previously shown [40-42]. The Bayesian and ML approaches for *L. longipalpis* collected in Bodocó, Caririaçu and Sobral indicate the presence of two genetically separated populations, and that each collected sample belongs to either 1S or 2S morphotype. Our analyses also indicated that these two genetically distinct populations are related to abdominal spots, as previously suggested for sympatric populations of *L. longipalpis* [21-23].

Analysis the 525 bp fragment of *per* in sand flies from Sobral showed greater separation between the two morphotypes, even when genetic distance methodology was used (Additional file 1: Figure S1). A Neighbor-joining tree added to the population genetic structure analyses confirmed the presence of cryptic species occurring in sympatry in Sobral, as noted by Bauzer et al. [23]. In that case, the presence of a haplotype shared by distinct morphotypes was observed. In contrast, our analyses using a greater number of polymorphisms revealed patterns of total association between phenotypes and genotypes [22,43].

As the number of shared haplotypes observed was lower in the 525 bp fragment of *per* in comparison to the 266 bp fragment (15 shared haplotypes versus 26, Additional file 2: Table S1), it was suggestive of the ability of the 525 bp *per* to detect greater genetic variation in *L. longipalpis* populations. Analyses by AMOVA of the results obtained with the 525 bp fragment confirmed a greater variation between phenotypically distinct groups characterized by the association of a pattern of abdominal spots and the genetic marker not previously reported for *L. longipalpis* populations.

In Sobral, *L. longipalpis* is described as distinct populations of two sympatric species, and commonly separated by the 1S and 2S phenotypes. Genetic markers, copulatory sounds, and sex pheromones have been characterized for each of the *L. longipalpis* populations found in Sobral and have been used to confirm the separation conferred by the 1S/2S phenotypes [19,23,44,45]. Conversely, *L. longipalpis* collected in the localities of Bodocó and Caririaçu are, according to our data, two genetically distinct populations, and the results observed, match the pattern of abdominal spots described in the sympatric populations [22,23,43]. Thus, as *L. longipalpis* from Bodocó and Caririaçu exhibit the same phenotypic and genetic patterns of populations found in Sobral, it is likely that these two populations also share chemical and behavioral characteristics similar to what was previously described for the *L. longipalpis* found in Sobral.

Our group previously reported on a secondary contact between *L. longipalpis* populations that were separated by the original course of the São Francisco River [46]. This secondary contact between the 1S populations of the Brazilian SE and NE may have promoted the genetic diversity of 1S. It also further reinforced the hypothesis that 2S actually derives from 1S, in accordance to the maximum likelihood tree.

## Conclusion

Understanding the complex population genetics of *L. longipalpis* and its pattern of distribution is critical in areas with high endemicity for the transmission of visceral leishmaniasis, such as the current situation in the state of Ceará in Brazil. The genetic analyses using the 525 bp fragment of the *per* gene revealed for the first time a moderate geographical structuring between the *L. longipalpis* populations, and a significant variability with regards to the 1S and 2S phenotypes. The results presented here also underscore the importance of the abdominal spots for the diagnosis of cryptic species of sympatric populations of the *L. longipalpis* complex in Brazil, and the use of *per* as an important barcode marker to populations of *L. longipalpis*. Further confirmation of the fixed SNPs identified in *per* and its application as taxonomic markers to differentiate sympatric *L. longipalpis* populations across Brazil is warranted.

## Ethical approval

Ethical approval was not required for the current study.

## Additional files

Additional file 1: Figure S1. Neighbor-joining phylogenetic tree of the *L. longipalpis*. Tree was constructed using the 525 bp fragment of the gene *period* obtained by PCR amplification of DNA extracted from *L. longipalpis* collected in Sobral, State of Ceara, Brazil. The tree shows the two phenotypic forms (1S and 2S) with bootstrap value of 67%. The parameters used for the reconstruction of the cladogram were the same as in Bauzer et al. [23]. Additional file 2: Table S1. Haplotypes frequency. Frequency of haplotypes generated with the fragment of 266 and 525 base pairs.
