# Supplementary material for: Genetic structuring and fixed polymorphisms in the gene period among natural populations of Lutzomyia longipalpis in Brazil
Source: Parasit Vectors. 2015 Apr 1;8:193. doi: 10.1186/s13071-015-0785-6 (PMC4409732; doi:10.1186/s13071-015-0785-6)
Supplement: Additional file 2: Table S1. — Haplotypes frequency. Frequency of haplotypes generated with the fragment of 266 and 525 base pairs. [file 13071_2015_785_MOESM2_ESM.docx]

Additional file 2: Table S1: Haplotype frequency of *Lutzomyia longipalpis* from three localities Ceará State, Brazil.

| Haplotype |  | Frequence | Sequences | | | | | | | | |
| --- | --- | --- | --- | --- | --- | --- | --- | --- | --- | --- | --- |
| Hap_2: | 1S | 8 | BOD1S02 | BOD1S08 | BOD1S11 | BOD1S16 | BOD1S18 | BOD1S22 | BOD1S24 | BOD1S31 |  |
| Hap_4: | 1S | 7 | BOD1S04 | BOD1S25 | BOD1S29 | SOB1S21 | SOB1S25 | SOB1S33 | CAR1S30 |  |  |
| Hap_5: | 1S | 7 | BOD1S05 | BOD1S06 | BOD1S15 | BOD1S19 | BOD1S20 | BOD1S32 | CAR1S08 |  |  |
| Hap_7: | 1S | 5 | BOD1S09 | SOB1S06 | SOB1S27 | CAR1S04 | CAR1S12 |  |  |  |  |
| Hap_10: | 1S | 3 | BOD1S21 | SOB1S22 | CAR1S25 |  |  |  |  |  |  |
| Hap_13: | 1S | 3 | BOD1S28 | BOD1S30 | CAR1S03 |  |  |  |  |  |  |
| Hap_45: | 1S | 3 | SOB1S19 | CAR1S05 | CAR1S15 |  |  |  |  |  |  |
| Hap_1: | 1S | 2 | BOD1S01 | BOD1S26 |  |  |  |  |  |  |  |
| Hap_31: | 1S | 2 | SOB1S02 | SOB1S05 |  |  |  |  |  |  |  |
| Hap_40: | 1S | 2 | SOB1S13 | SOB1S35 |  |  |  |  |  |  |  |
| Hap_53: | 1S | 2 | SOB1S31 | CAR1S11 |  |  |  |  |  |  |  |
| Hap_64: | 1S | 2 | CAR1S14 | CAR1S24 |  |  |  |  |  |  |  |
| Hap_68: | 1S | 2 | CAR1S20 | CAR1S28 |  |  |  |  |  |  |  |
| Hap_3: | 1S | 1 | BOD1S03 |  |  |  |  |  |  |  |  |
| Hap_6: | 1S | 1 | BOD1S07 |  |  |  |  |  |  |  |  |
| Hap_8: | 1S | 1 | BOD1S13 |  |  |  |  |  |  |  |  |
| Hap_9: | 1S | 1 | BOD1S14 |  |  |  |  |  |  |  |  |
| Hap_11: | 1S | 1 | BOD1S23 |  |  |  |  |  |  |  |  |
| Hap_12: | 1S | 1 | BOD1S27 |  |  |  |  |  |  |  |  |
| Hap_14: | 1S | 1 | BOD1S33 |  |  |  |  |  |  |  |  |
| Hap_30: | 1S | 1 | SOB1S01 |  |  |  |  |  |  |  |  |
| Hap_32: | 1S | 1 | SOB1S03 |  |  |  |  |  |  |  |  |
| Hap_33: | 1S | 1 | SOB1S04 |  |  |  |  |  |  |  |  |
| Hap_34: | 1S | 1 | SOB1S07 |  |  |  |  |  |  |  |  |
| Hap_35: | 1S | 1 | SOB1S08 |  |  |  |  |  |  |  |  |
| Hap_36: | 1S | 1 | SOB1S09 |  |  |  |  |  |  |  |  |
| Hap_37: | 1S | 1 | SOB1S10 |  |  |  |  |  |  |  |  |
| Hap_38: | 1S | 1 | SOB1S11 |  |  |  |  |  |  |  |  |
| Hap_39: | 1S | 1 | SOB1S12 |  |  |  |  |  |  |  |  |
| Hap_41: | 1S | 1 | SOB1S15 |  |  |  |  |  |  |  |  |
| Hap_42: | 1S | 1 | SOB1S16 |  |  |  |  |  |  |  |  |
| Hap_43: | 1S | 1 | SOB1S17 |  |  |  |  |  |  |  |  |
| Hap_44: | 1S | 1 | SOB1S18 |  |  |  |  |  |  |  |  |
| Hap_46: | 1S | 1 | SOB1S20 |  |  |  |  |  |  |  |  |
| Hap_47: | 1S | 1 | SOB1S23 |  |  |  |  |  |  |  |  |
| Hap_48: | 1S | 1 | SOB1S24 |  |  |  |  |  |  |  |  |
| Hap_49: | 1S | 1 | SOB1S26 |  |  |  |  |  |  |  |  |
| Hap_50: | 1S | 1 | SOB1S28 |  |  |  |  |  |  |  |  |
| Hap_51: | 1S | 1 | SOB1S29 |  |  |  |  |  |  |  |  |
| Hap_52: | 1S | 1 | SOB1S30 |  |  |  |  |  |  |  |  |
| Hap_54: | 1S | 1 | SOB1S32 |  |  |  |  |  |  |  |  |
| Hap_55: | 1S | 1 | SOB1S34 |  |  |  |  |  |  |  |  |
| Hap_56: | 1S | 1 | SOB1S36 |  |  |  |  |  |  |  |  |
| Hap_57: | 1S | 1 | CAR1S01 |  |  |  |  |  |  |  |  |
| Hap_58: | 1S | 1 | CAR1S02 |  |  |  |  |  |  |  |  |
| Hap_59: | 1S | 1 | CAR1S06 |  |  |  |  |  |  |  |  |
| Hap_60: | 1S | 1 | CAR1S07 |  |  |  |  |  |  |  |  |
| Hap_61: | 1S | 1 | CAR1S09 |  |  |  |  |  |  |  |  |
| Hap_62: | 1S | 1 | CAR1S10 |  |  |  |  |  |  |  |  |
| Hap_63: | 1S | 1 | CAR1S13 |  |  |  |  |  |  |  |  |
| Hap_65: | 1S | 1 | CAR1S17 |  |  |  |  |  |  |  |  |
| Hap_66: | 1S | 1 | CAR1S18 |  |  |  |  |  |  |  |  |
| Hap_67: | 1S | 1 | CAR1S19 |  |  |  |  |  |  |  |  |
| Hap_69: | 1S | 1 | CAR1S16 |  |  |  |  |  |  |  |  |
| Hap_70: | 1S | 1 | CAR1S21 |  |  |  |  |  |  |  |  |
| Hap_71: | 1S | 1 | CAR1S26 |  |  |  |  |  |  |  |  |
| Hap_72: | 1S | 1 | CAR1S27 |  |  |  |  |  |  |  |  |
| Hap_73: | 1S | 1 | CAR1S29 |  |  |  |  |  |  |  |  |
| Hap_23: | 2S | 11 | BOD2S16 | BOD2S20 | BOD2S21 | BOD2S27 | BOD2S33 | SOB2S04 | CAR2S04 | CAR2S18 | CAR2S19 |
| Hap_20: | 2S | 9 | BOD2S06 | BOD2S18 | SOB2S08 | SOB2S14 | SOB2S17 | SOB2S18 | CAR2S24 | CAR2S29 | CAR2S32 |
| Hap_18: | 2S | 8 | BOD2S04 | BOD2S07 | BOD2S13 | BOD2S29 | SOB2S02 | SOB2S23 | SOB2S28 | CAR2S30 |  |
| Hap_77: | 2S | 4 | SOB2S06 | CAR2S01 | CAR2S02 | CAR2S25 |  |  |  |  |  |
| Hap_75: | 2S | 3 | SOB2S03 | CAR2S05 | CAR2S06 |  |  |  |  |  |  |
| Hap_79: | 2S | 3 | SOB2S10 | SOB2S21 | CAR2S07 |  |  |  |  |  |  |
| Hap_86: | 2S | 3 | SOB2S26 | CAR2S14 | CAR2S27 |  |  |  |  |  |  |
| Hap_17: | 2S | 2 | BOD2S03 | BOD2S17 |  |  |  |  |  |  |  |
| Hap_22: | 2S | 2 | BOD2S14 | CAR2S08 |  |  |  |  |  |  |  |
| Hap_26: | 2S | 2 | BOD2S23 | SOB2S16 |  |  |  |  |  |  |  |
| Hap_85: | 2S | 2 | SOB2S24 | CAR2S03 |  |  |  |  |  |  |  |
| Hap_88: | 2S | 2 | CAR2S10 | CAR2S13 |  |  |  |  |  |  |  |
| Hap_89: | 2S | 2 | CAR2S11 | CAR2S21 |  |  |  |  |  |  |  |
| Hap_15: | 2S | 1 | BOD2S01 |  |  |  |  |  |  |  |  |
| Hap_16: | 2S | 1 | BOD2S02 |  |  |  |  |  |  |  |  |
| Hap_19: | 2S | 1 | BOD2S05 |  |  |  |  |  |  |  |  |
| Hap_21: | 2S | 1 | BOD2S09 |  |  |  |  |  |  |  |  |
| Hap_24: | 2S | 1 | BOD2S19 |  |  |  |  |  |  |  |  |
| Hap_25: | 2S | 1 | BOD2S22 |  |  |  |  |  |  |  |  |
| Hap_27: | 2S | 1 | BOD2S24 |  |  |  |  |  |  |  |  |
| Hap_28: | 2S | 1 | BOD2S28 |  |  |  |  |  |  |  |  |
| Hap_29: | 2S | 1 | BOD2S32 |  |  |  |  |  |  |  |  |
| Hap_74: | 2S | 1 | SOB2S01 |  |  |  |  |  |  |  |  |
| Hap_76: | 2S | 1 | SOB2S05 |  |  |  |  |  |  |  |  |
| Hap_78: | 2S | 1 | SOB2S07 |  |  |  |  |  |  |  |  |
| Hap_80: | 2S | 1 | SOB2S11 |  |  |  |  |  |  |  |  |
| Hap_81: | 2S | 1 | SOB2S12 |  |  |  |  |  |  |  |  |
| Hap_82: | 2S | 1 | SOB2S15 |  |  |  |  |  |  |  |  |
| Hap_83: | 2S | 1 | SOB2S20 |  |  |  |  |  |  |  |  |
| Hap_84: | 2S | 1 | SOB2S22 |  |  |  |  |  |  |  |  |
| Hap_87: | 2S | 1 | CAR2S09 |  |  |  |  |  |  |  |  |
| Hap_90: | 2S | 1 | CAR2S15 |  |  |  |  |  |  |  |  |
| Hap_91: | 2S | 1 | CAR2S16 |  |  |  |  |  |  |  |  |
| Hap_92: | 2S | 1 | CAR2S17 |  |  |  |  |  |  |  |  |
| Hap_93: | 2S | 1 | CAR2S20 |  |  |  |  |  |  |  |  |
| Hap_94: | 2S | 1 | CAR2S22 |  |  |  |  |  |  |  |  |
| Hap_95: | 2S | 1 | CAR2S28 |  |  |  |  |  |  |  |  |
| Hap_96: | 2S | 1 | CAR2S31 |  |  |  |  |  |  |  |  |
